# A Means of Emptying the Bladder

**Published:** 1910-11

**Authors:** 


### A Means of Emptying the Bladder.

The bladder, when partially paralyzed from parturition, or any other cause, can always be made to empty itself perfectly by throwing a large amount of very warm water into the bowel, thereby doing away with the necessity of using a catheter—a most important consideration, particularly when the patient lives at a distance from the doctor. After difficult and protracted labors, I have been obliged to use the catheter every day for weeks at a time, which was annoying to the patient and inconvenient to myself. Since using the above recommended plan, I have had no trouble in this direction, the bowel and the bladder emptying themselves at the same time.—*Southern California Practitioner.*

